# Antimicrobial resistance profiles and virulence genotyping of *Salmonella enterica* serovars recovered from broiler chickens and chicken carcasses in Egypt

**DOI:** 10.1186/s12917-019-1867-z

**Published:** 2019-04-27

**Authors:** Rasha Elkenany, Mona Mohieldin Elsayed, Amira I. Zakaria, Shimaa Abd- El-Salam El-sayed, Mohamed Abdo Rizk

**Affiliations:** 1 Department of Bacteriology, Mycology and Immunology, Faculty of Veterinary Medicine, Mansoura University, Mansoura, 35516 Egypt; 2 Department of Hygiene and Zoonosis, Faculty of Veterinary Medicine, Mansoura University, Mansoura, 35516 Egypt; 3 Department of Food Hygiene and Control, Faculty of Veterinary Medicine, Mansoura University, Mansoura, 35516 Egypt; 4 Department of Biochemistry and Chemistry of Nutrition, Faculty of Veterinary Medicine, Mansoura University, Mansoura, 35516 Egypt; 5 Department of Internal Medicine and Infectious Diseases, Faculty of Veterinary Medicine, Mansoura University, Mansoura, 35516 Egypt

## Abstract

**Background:**

This study aimed to survey the prevalence, antimicrobial resistance, and virulence-associated genes of *Salmonella enterica* recovered from broiler chickens and retail shops at El-Sharkia Province in Egypt. *Salmonella* virulence factors were determined using the polymerase chain reaction assays targeting the *invA, csgD, hilC, bcfC, stn, avrA, mgtC, ompF, sopE1* and *pefA* genes.

**Results:**

One hundred tweenty out of 420- samples from broiler chickens’ cloacal swabs, farm environmental samples, and freshly dressed whole chicken carcasses were positive *Salmonella* species. The isolates were serotyped as *S.* Enteritidis as the most dominant serotypes*.* Interestingly, none of the isolates were resistant to imipenem. The multidrug resistance was determined in 76.7% of the isolates with multidrug antibiotic resistance index of 0.2–0.6. Eight virulence genes (*invA, csgD, hilC, stn, bcfC, mgtC, avrA,* and *ompf*) were characterized among 120 *S. enterica* isolates with variable frequencies, while *sopE1*and *pefA* genes that were completely absent in all isolates. Based on the combination of presence and absence of virulence genes, the most common genetic profile (P7, 30%) was *invA* and *csgD* genes.

**Conclusion:**

*S*. Enteritidis and *S*. Typhimurium were the most common identified serotypes in the examined sources. Circulation of such strains in broiler farms required introducing special biosecurity and biocontrol measures for control of *Salmonella*. Such measures might limit the adverse effects of antibiotics and ensure the safety of the environment and animal-derived food.

**Electronic supplementary material:**

The online version of this article (10.1186/s12917-019-1867-z) contains supplementary material, which is available to authorized users.

## Background

*Salmonella enterica* is one of the major foodborne pathogens leading to worldwide human gastroenteritis [1]. *S.* Enteritidis was considered the most frequent one followed by S. Typhimurium isolated from human worldwide [2]. Of note, poultry is usually incriminated in outbreaks of human salmonellosis [2]. Therefore, the detection of *Salmonella* species in poultry production chain especially at the farms level is an issue of large concern. Furthermore, the resistance of some *Salmonella* serotype to multiple antibiotics [3], makes the study of the antibiotic susceptibility profile and its ecology of this zoonotic pathogen has a great priority. Indeed, the widespread misapplication and overuse of antimicrobial agents in food animal production have contributed to the development of antimicrobial resistant pathogens such as *Salmonella* that has emerged as a major public health implication [2].

Virulence gene encodes products that aid the organisms to interact with the host cells [4]. To that end, numerous virulence genes are incriminated in the pathogenesis of salmonellosis [5]. These genes are clustered within *Salmonella* pathogenicity islands (SPIs)-1 and − 21 (SPI-1 to SPI-21) and participate in the adhesion and invasion of the pathogen to the host as *inv* gene or help in the pathogen survival within the host like *mgtC5* gene [6]. Serovars like *S.* Typhimurium also harbor self-transmissible virulence plasmid-encoded fimbriae (*pef*) fimbrial operon [7]. The enterotoxin (*stn*) gene was demonstrated as a suitable PCR target for detection of *Salmonella* strains [8]. In fact, previous studies had demonstrated the molecular characterization and antibiotic resistance of *Salmonella* serovars isolated from chickens farms in Kafr El-Sheikh Province, Egypt during 2014–2015 [9] and Sharkia Province [5] during 2009–2010 in Northern Egypt. While these studies used only clinical samples collected from different organs of broiler flocks without highlighting the prevalence of the infection in the surrounding farm environment and workers hand. Additionally, El-Sharkawy et al. [9] and Ammar et al. [5] didn’t investigate selected genes (*csgD, hilC, bcfC, mgtC, avrA*, *ompf* and *pefA*) and selected antimicrobial agents (cefaclor, cefotaxime, cefepime and imipenem). Therefore, this study was set out to determine the prevalence of various *Salmonella* serovars in broiler chickens, chicken carcasses, drinking water, feed, litter, fans swabs and workers hand collected from broiler chickens farms at El-Sharkia province in Egypt. Moreover, the present study highlighted the antimicrobial resistance profiles of *Salmonella* serotypes using 11- antimicrobial agents (amoxicillin-clavulanic acid (AMC), ampicillin (AMP), cefaclor (CEC), cefotaxime (CTX), cefepime (FEP), doxycycline (DO), ciprofloxacin (CIP), imipenem (IPM), streptomycin (S), chloramphenicol (C) and trimethoprim-sulfamethoxazole (SXT) commonly used in human and veterinary medicines. Additionally, the pathogenic potential of recovered *Salmonella* serovars was assessed in the present study using virulotyping PCR assay targeting the *invA, csgD, hilC, bcfC, stn, avrA, mgtC, ompF, sopE1* and *pefA* gene sequences. To the best of our knowledge, this is the first study that determines the distribution of *csgD, hilC* and *ompF* genes in *Salmonella* isolates from chickens in Egypt.

## Results

### Prevalence and serotypes of isolated *Salmonella enterica*

The prevalence and serotypes of *Salmonella enterica* were screened in the present study in samples collected from chickens’ cloacal swabs, farm environmental samples and freshly dressed whole chicken carcasses at El-Sharkia province in Egypt. Of 420 samples, the *Salmonella* species were identified in 120 (28.6%) samples (Table 1). The *Salmonella* strains were observed in 32% (48/150) of cloacal swabs, 22% (22/100) of environmental samples (2- samples from the litter, 8- samples from the drinking water, 8- samples from the feed, 1- sample from the workers hand and 3- samples from the fans swabs) and 29.4% (50/170) of whole chicken carcasses (Table 1). In general, *S.* Enteritidis (11.4%) was the most common identified serotypes followed by *S.* Typhimurium (8.6%), *S.* Kentucky and *S.* Molade (2.85% each), *S.* Bargny (1.4%), *S.* Inganda (0.95%) and *S.* Infantis (0.48%) (Table 1). The identified serovars from cloacal swabs were *S.* Enteritidis (20 isolates), *S.* Typhimurium (19 isolates), *S.* Kentucky (1 isolates), *S.* Molade (6 isolates) and *S.* Bargny (2 isolates). While, the isolated strains from whole chicken carcasses were *S.* Enteritidis (18 isolates), *S.* Typhimurium (11 isolates), *S.* Kentucky (8 isolates), *S.* Molade (6 isolates), *S.* Bargny (2 isolates), *S.* Inganda (4 isolates), *S.* Infantis (one isolate). The isolated serovars from environmental samples were *S.* Enteritidis (10 isolates), *S.* Typhimurium (6 isolates), *S.* Kentucky (3 isolates), *S.* Bargny (2 isolates) and *S.* Infantis (one isolate) (Table 1). The results indicated that *S.* Enteritidis was the most dominant *Salmonella* serotype in chicken in El-Sharkia Province in Egypt.

**Table 1:** Distribution of *Salmonella* serovars in study samples (*n* = 120)

Serotypes	Antigenic formula	Sample type (n)			Total (*n* = 420)
		Broiler chicken farms		Retail shops	
		Cloacal swabs (*n* = 150)	Environmental samples (*n* = 100)	Chicken carcass (*n* = 170)	
*S.* Enteritidis	O:1,9,12; H_1_:g,m; H_2_:-	20	10	18	48 (11.4%)
*S.* Typhimurium	O:1,4,5,12; H_1_:I;H_2_:1,2	19	6	11	36 (8.6%)
*S.*Kentucky	O:8,20; H_1_:I; H_2_:Z_6_	1	3	8	12 (2.85%)
*S.*Molade	O:8,20;H1:Z10;H2:Z6	6	–	6	12 (2.85%)
*S.* Bargny	O:8,20;H1:i;H2:1,5	2	2	2	6 (1.4%)
*S.* Inganda	O:6,7;H1:Z10;H2:1,5	–	–	4	4 (0.95%)
*S.* Infantis	O:6,7;H1:r;H2:1,5	–	1	1	2 (0.48%)
Total		48 (32%)	22 (22%)	50 (29.4%)	120 (28.6%)

### Antimicrobial resistance and distribution among differently identified *Salmonella* serovars

Variable rates of resistance of *Salmonella* serotypes were observed against 11 different types of antimicrobials. The antimicrobial susceptibility testing revealed absolute resistance to SXT (100%), AMP, AMC (68.3% each), S (65%), DO (40%) and CEC (36.7%). On the other hand, lower rates of resistance were observed for CIP, (10%), CTX and FEP (13.3% each) and C (16.7%). Interestingly, none of the isolates were resistant to IPM (Table 2). *Salmonella* isolates were showed resistant to two and up to seven antimicrobial agents (Table 3). In addition, multidrug resistance (MDR) to three or more antimicrobial classes was detected in 92 out of 120 (76.7%) isolates with multidrug antibiotic resistance index (MARI) of 0.2–0.6 (Table 3). *Salmonella* serovars in this study demonstrated 11 different MDR patterns (Table 3), reflecting the high prevalence of MDR among Salmonella isolates in the surveyed Province.

**Table 2:** Antimicrobial resistance profiles of isolated *Salmonella* serovars

Serovars (n)	Antimicrobial resistance										
	SXT	AMP	AMC	S	DO	CEC	C	FEP	CTX	CIP	IPM
*S.* Enteritidis (48)	48	34	42	28	24	20	12	10	6	6	0
*S.* Typhimurium (36)	36	18	22	22	14	18	6	4	4	2	0
*S.* Kentucky (12)	12	12	10	10	8	4	2	2	6	4	0
*S.* Molade (12)	12	8	6	8	2	2	0	0	0	0	0
*S.* Bargny (6)	6	4	2	4	0	0	0	0	0	0	0
*S.* Inganda (4)	4	4	0	4	0	0	0	0	0	0	0
*S.* Infantis (2)	2	2	0	2	0	0	0	0	0	0	0
Total (120)	120	82	82	78	48	44	20	16	16	12	0
^a^ Resistant %	100%	68.%	68.%	65%	40%	36.7%	16.7%	13.3%	13.3%	10%	0
^a^ Intermediate %	0	4.2%	0	8.3%	10.8%	12.5%	16.7%	0	5.8%	0	0
^a^ Susceptible %	0	27.5%	36.7%	26.7%	49.2%	50.8%	66.7%	86.7%	80.8%	90%	100%

*AMC* Amoxicillin-clavulanic acid, *AMP* Ampicillin, *CEC* Cefaclor, *CTX* Cefotaxime, *FEP* Cefepime, *DO* Doxycycline, *CIP* Ciprofloxacin, *IPM* Imipenem, *S* Streptomycin, *C* Chloramphenicol and *SXT* Trimethoprim sulfamethoxazole

^a^The percentage of the total number of isolates resistant, intermediate, or susceptible for a particular antimicrobial is indicated in the last three rows below each antimicrobial

**Table 3:** Distribution of antibiotic resistance rates of *Salmonella* isolates

Antibiotic pattern profile	Antibiotics	No. of isolates	Percentage of resistant isolates (%)	No. of resistance antibiotics	MARI (%)
1	SXT, AMP,AMC,S, DO,CEC,C	10	8.3	7	0.6
2	SXT,AMP,AMC,S,CEC,C,FEP	6	5	7	0.6
3	SXT,AMP,AMC,S,DO,CTX,CIP	6	5	7	0.6
4	SXT, AMP,AMC,S, DO,CEC	8	6.7	6	0.6
5	SXT,AMP,AMC,CEC,FEP,CTX	10	8.3	6	0.6
6	SXT, AMP,AMC,S,DO	10	8.3	5	0.5
7	SXT, AMP,S,DO,CIP	6	5	5	0.5
8	SXT,S, DO,CEC	10	8.3	4	0.4
9	SXT,AMP,AMC,C	4	3.3	4	0.4
10	SXT, AMP,S	22	18.3	3	0.3
11	SXT,AMC	28	23.3	2	0.2

*AMC* Amoxicillin-clavulanic acid, *AMP* Ampicillin, *CEC* Cefaclor, *CTX* Cefotaxime, *FEP* Cefepime, *DO* Doxycycline, *CIP* Ciprofloxacin, *IPM* Imipenem, *S* Streptomycin, *C* Chloramphenicol, *SXT* Trimethoprim sulfamethoxazole, and *MARI* Multidrug antibiotic resistance index

### Distribution of virulence genes among *Salmonella* serovars

PCR targeting 10 virulence genes (*invA, csgD, hilC, bcfC, stn, avrA, mgtC, ompF, sopE1* and *pefA*) were performed in this study to detect the identified *Salmonella* serovars virulence (Additional file 1: Figures S1-S3). Generally speaking, all *Salmonella* isolates showed at least two virulence-associated genes (Table 4). Of note, *invA* gene (genus specific gene) was detected in 100% (120/120) of the isolates. On the contrary, *sopE1* and *pefA genes* were completely absent in all isolates (Table 4). *csgD* and *hilC* genes were investigated in 90% (108/120) and 60% (72/120) of the isolates, respectively. The genes *bcfC* and *stn* were simultaneously detected in 40% (48/120) of the isolates and 30% (36/120) of the isolates were positive for *avrA* (located on SPI-1) and *mgtC* (carried on SPIs) genes. Whilst, the *ompF* gene was present in 20% (24/120) of the isolates (Table 4). Different frequencies of virulence genes among various serovars were detected with the absence of *stn, avrA, mgtC* and *ompF* genes among the isolates; *S.* Molade, *S.* Bargny, *S.* Inganda and *S.* Infantis (Table 4).

**Table 4:** Distribution of virulence genes among different *Salmonella* serovars

Serovars (n)	Virulence genes (n)									
	*invA*	*csgD*	*hilC*	*bcfC*	*Stn*	*avrA*	*mgtC*	*ompF*	*SopE1*	*pefA*
*S.* Enteritidis (48)	48	44	42	28	36	20	18	6	0	0
*S.* Typhimurium (36)	36	36	6	16	12	14	16	16	0	0
*S.* Kentucky (12)	12	10	10	2	0	2	2	2	0	0
*S.* Molade (12)	12	10	4	2	0	0	0	0	0	0
*S.* Bargny (6)	6	6	6	0	0	0	0	0	0	0
*S.* Inganda (4)	4	0	4	0	0	0	0	0	0	0
*S.* Infantis (2)	2	2	0	0	0	0	0	0	0	0
Total (%)	120 (100%)	108 (90%)	72 (60%)	48 (40%)	48 (40%)	36 (30%)	36 (30%)	24 (20%)	0	0

Based on the combination of present and absent virulence genes, the *Salmonella* isolates were divided into seven different genetic profiles that were devoid of *SopE1* and *pefA* (Table 5). In order to facilitate the analysis, these profiles were named P1- P7. Regarding the profiles, among the 120- analyzed isolates, 10% (12/120) were categorized as P1 (positive for all genes except SopE1 and pefA), 10% as P2 (*hilC* absent), 10% as P3 (*ompF* absent), 10% as P4 (*avrA, mgtC* and *ompF* absent), 20% as P5 (*invA, hilC* and *csgD* genes only present), 10% as P6 (*invA* and *hilC* only present) and 30% as P7 (*invA* and *csgD* only present) (Table 5).

**Table 5:** Virulence profile of *Salmonella* serovars isolated in this study

Genetic profile	Virulence genes	No. of isolates (%)
P1	*invA,csgD,hilC,bcfC,stn,avrA,mgtC,ompF*	12 (10%)
P2	*invA,csgD,bcfC,stn,avrA,mgtC,ompF*	12 (10%)
P3	*invA,csgD, hilC,bcfC,stn,avrA,mgtC*	12 (10%)
P4	*invA,csgD, hilC,bcfC,stn*	12 (10%)
P5	*invA,csgD, hilC*	24 (20%)
P6	*invA,hilC*	12 (10%)
P7	*invA,csgD*	36 (30%)

### Relationship between antimicrobial resistance pattern and virulence determinants

The presence of virulence determinants *(invA, csgD, hilC, bcfC, stn, avrA, mgtC* and *ompF)* in different *Salmonella* serovars recovered from cloacal swabs, farm environment and whole chicken carcasses samples exhibited various antimicrobial resistance patterns as shown in Additional file 2: Table S2. A detailed analysis displayed associations of resistance phenotypes with potential virulence genes.

## Discussion

In the present study, seven *Salmonella* serovars were identified from examined samples with a notably high prevalence of *S.* Enteritidis (11.4%) and *S.* Typhimurium (8.6%). These results were in concordance with those observed in dead and diseased chickens by Rabie et al. [10], Ammar et al. [5] in Egypt and Borges et al. [11] in Brazil. Moreover, a higher isolation rate of *Salmonella* spp. was detected in broiler chickens’ cloacal swabs followed by whole chicken carcasses and farm environmental samples. Both drinking water and feeding are considered the main sources of contamination inside the farms. In contrast to ours, the isolation rate of *Salmonella* spp. in chickens’ wastewater (20%) was higher than those (9.2%) detected in the whole chicken carcass in a study performed by Nwiyi et al. [12]. Such higher prevalence of *Salmonella* spp. in the whole chicken carcasses might be attributed to low slaughter hygiene, cross-contamination of products at different stages of chicken dressing and preparation in the retail shops at El-Sharkia province, Egypt. Also, isolation of *Salmonella enterica serovars* with high percent from broiler chicken farms necessitated the application of biosecurity program inside the farms beside using alternatives to the antibiotics such as bacteriophages or herbal extracts. Such alternative therapeutic interventions may help in cutting the cycle of horizontal transmission of *Salmonella* to broiler carcasses.

Increasing rates of antimicrobial resistance among *Salmonella* is a growing healthcare problem that needs to be monitored continuously. Our study indicated that all isolated *Salmonella* strains exhibited absolute resistance (100%) against trimethoprim-sulfamethoxazole, indicating the limited therapeutic value of this antibiotic to poultry. Higher rates of resistance were observed to extended spectrum penicillin [ampicillin and amoxicillin-clavulanic acid], streptomycin, cefaclor, and doxycycline. These antimicrobial resistances of *Salmonella* spp. to extended spectrum penicillin, streptomycin, cefaclor, and doxycycline were cited previously in Egypt [5, 13], and in Turkey [14]. Interestingly, the resistance of *Salmonella* spp. to cefaclor, a second-generation cephalosporin antibiotic was detected in the present study (36.7%) which is higher than those (14.5%) recently detected in Saudi Arabia by Abo-Amer and Shobrak [15]. In the current study, 13.3% of *Salmonella* spp. isolated showed resistance to cefepime, a fourth-generation cephalosporin antibiotic, which was consistent to a previous observation by Mir et al. [16] in poultry in India. In an astonishing way, no resistance was detected from *Salmonella* serovars to imipenem. Such absence of resistance to imipenem might be attributed to the fact that there was no history of using this antimicrobial candidate for the prevention or treatment in commercial chickens farms in EI-Sharkia Province. Therefore, other studies are warranted to evaluate the inhibitory effect of imipenem against *Salmonella* spp. in vivo.

In fact, MDR pathogens create a difficulty in the treatment of human and animal illnesses and MDR strains of *Salmonella* have been associated with high morbidity, compared to susceptible strains [17]. Unfortunately, results obtained in the current study revealed MDR against three or more antibiotics in 76.7% of isolates with MARI ranged from 0.2 to 0.6. MARI value lower than 0.2, is considered a low risk, while value higher than 0.2 indicates high risk [17]. This result was compatible with Chuanchuen et al. [18]*,* who isolated 70% of multiresistant *Salmonella* from poultry and swine with the most resistant pattern to ampicillin, chloramphenicol, streptomycin, sulfamethoxazole, tetracycline, and trimethoprim. The higher MARI value that was observed in the present study might be attributed to the widespread use of antibiotics in the locality in Egypt, the indiscriminate use of antibiotics either at recommended doses or at sub-therapeutic doses as feed additives to promote the growth of the poultry in developing countries. Subsequently, multi-drug resistant *Salmonellae* constituted a public health hazard and potentially affected the efficacy of medications in humans [19]. The increasing occurrence of *Salmonella* serovars resistant to sulfonamides, β-lactam, and aminoglycosides is considered alarming, as they are used for the treatment of invasive salmonellosis [13].

In the present study, well-recognized 10- virulence genes (*invA, csgD, hilC, bcfC, stn, avrA, mgtC, ompf, sopE1* and *pef*A) were screened using PCR assay. Considering the importance of their function, for the first time in Egypt, the prevalence of the *csgD, hilC,* and *ompF* genes were evaluated to find out whether these genes can be detected in *Salmonella* isolates or not. The investigated genes comprised *invA, hilC*, *avrA* and *mgtC* genes associated with SPIs, the biofilm-associated gene *csgD*, the fimbrial related gene *bcfC,* the *stn* gene involved in heat labile *Salmonella* enterotoxin production, the outer membrane porin F (*ompF*) gene as a major general diffusion porin, *sop*E1 gene encoding a translocate effector protein and *pef*A gene as plasmid-encoded fimbria were also investigated in this study.

The *csgD* gene is the master regulator of the biofilm matrix compounds of *Salmonella to* promote the survival of bacteria when they are exposed to unsuitable conditions and was widely distributed among *Salmonella* isolates (90%) in our study regardless of their serovars. The *hilC* gene is located on SPI-1 and modulates invasion gene expression [20]. Irrespective of their serovars, a *hilC* gene was detected in 60% of analyzed strains. Compared to previous investigations, *a hil*C gene was absent in all of the *Salmonella* isolates from poultry [21]*.*

The outer membrane porin (*ompF*) allows substrates across the membrane in Gram-negative organisms and does a non-specific cation prefer porin [22]. In this study, *ompF* gene was detected only in 20% of *Salmonella* isolates. While, a previous study [23] detected *ompF* gene in all 218- *Salmonella* strains surveyed in the USA. Interestingly, the isolates for the *sopE1* gene coded by SPI-5 were screened, and the results revealed its absence in all isolates as compared with 41.18% prevalence of *sopB* gene in the isolates obtained from the liver, heart, and spleen collected from freshly dead and diseased broiler chicken previously screened in Sharkia Province in Egypt during 2009–2010 [5]. Similar to our findings, Abd El- Tawab et al. [24] reported the absence of *sopE* gene in *Salmonella* isolates recovered from milk samples of cattle with clinical mastitis in Egypt. Additionally, a very low percentage (7.7%) of *sopE* gene was detected in the *Salmonella* isolates obtained from chicken hatchlings [25].

The ability of antimicrobial resistant *Salmonella* strains to produce invasive disease can be attributed to various virulence genes, and virulotyping rapidly allows the discrimination of isolates with diverse pathogenic potential [26]. Indeed, there are numerous factors incriminated in the antimicrobial resistance acquisition or dissemination in *Salmonella* species like the misuse of antibiotics, unregular sales and inappropriate prescription of antibiotics, the presence of mobile genetic elements in the organisms; plasmid DNA, transposons, integrons etc. [27]. The present study proved the spreading of antimicrobial resistance patterns and virulence determinants in the analyzed isolates. This finding is significant with respect to public health and had been previously reported in Egypt [5, 25]. In general, acquisition of the antimicrobial resistance affects the virulence in the invading bacteria through two alternative scenarios; increased resistance is accompanied by increased virulence (a positive effect) or increased antimicrobial resistance reduces the bacteria virulence (apparently negative effect) [28]. For *Salmonella* virulence, the resistance to aminoglycosides is associated with fitness cost in the *Salmonella* spp. [28]. Similarly, the resistance to fluoroquinolones has an implication in the fitness cost of *S. enterica* [29]*,* and a higher risk of invasive illness or death of *S.* Typhimurium [30]. The association between antibiotic resistance and virulence among *Salmonella* serovars happened due to the genetic determinants for the antibiotic resistance in addition to virulence genes could be harbored by the same transferable element [31, 32]. Generally speaking, specific pathogenicity genes (SPIs) are the main feature differentiate the pathogenic *Salmonella* spp. from the non-pathogenic ones and contribute to both natural and acquired resistance in *Salmonella* spp. [33]. The *invA, hilC, avrA* and *mgtC* genes that screened in the present study are associated with SPIs and were detected previously in the resistant strains [33, 34]. Additionally, the *omps* and *stn* genes are commonly distributed among the resistant *Salmonella* strains and have a global contribution for *Salmonella*-associated diseases in animal and human populations [34, 35].

## Limitations

It should be noted that there are some limitations to the present study. Although this is the first study addressing *csgD, hilC and ompF* genes in *Salmonella enterica* isolates using PCR in Egypt, it focused on chicken samples collected from only one province of Egypt and didn’t elucidate the antimicrobial resistance profiles and virulence genotyping of *Salmonella enterica* in other provinces. Therefore, additional studies are warranted to explore such profiles in other provinces of Egypt. The present study reported no resistance from *Salmonella* serovars to imipenem. However, further studies are required to confirm the potential of imipenem in the treatment of salmonellosis in chickens by evaluating the inhibitory effect of this candidate against *Salmonella* serovars isolated from different localities in Egypt and estimating the resistance of these isolates to imipenem. Moreover, future in-depth studies are necessary for analyzing the synergistic or an antagonistic effect of imipenem when used in combination with commonly used anti-*Salmonella* drugs and to determine the best effective composition ratio for the growth inhibition of *Salmonella* for clinical application. These drugs might be more effective if used as a part of a combination therapy rather than a single therapy.

## Conclusions

Multidrug resistance (MDR) and virulent *Salmonella* serovars is highly prevalent in broiler chickens, chicken carcass and farm environment in Egypt. Serotyping of recovered *Salmonella*, clarified predominance of *S.* Enteritidis and *S.* Typhimurium in examined sources, but other five serovars were also encountered. These findings clearly demonstrated the high prevalence of MDR *Salmonella* serovars that indicated alarming in the veterinarian therapeutic treatment. The virulotyping verified the variety in number and distribution of different virulence-associated genes among screened *Salmonella* serovars and provided additional evidence on the risk of virulent salmonellosis posted from chickens. Finally, the obtained data provide a more accurate profile for understanding the dangerous spread of virulence genotypes and antibiotic resistance in *Salmonella* serovars. Such data imposes planning and application of biosecurity programs in addition to the establishment of bio-control measures to control *Salmonella* infection inside broiler chicken farms.

## Methods

### Sample collection and preparation

A total of 420- broiler chickens’ cloacal swabs, farm environmental samples and freshly dressed chicken carcasses (humanly euthanasia using physical method; cervical dislocation) were randomly collected from five small scale broiler chicken farms at 3 weeks of growing cycle and five retail shops at El-Sharkia Province, Egypt during summer 2017 and used in this study. In details, 100 samples were collected from the farm environment (20 samples per farm) including drinking water (25 ml), feed (25 g), litter (25 g), fans and workers hand (4 samples for each type), 150 cloacal swabs (30 samples per farm) and 170 freshly dressed whole chicken carcasses samples (34 samples per shop) from outer skin were obtained from retail shops. All collected samples were subjected separately into a sterile impermeable labeled polyethylene bag (Thomas Scientific, USA), and transferred within 1 h in an icebox at 4 °C for bacteriological analysis. All collected samples (25 g or 25 ml) were aseptically placed into sterile Difco-buffered peptone water (BPW) (225 ml) tubes (Oxoid, UK) and pre-enriched at 37 °C for 24 h [36].

### Isolation and identification of *Salmonella*

Each pre-enriched homogenate (1 ml) was aseptically added to 10 ml of Rappaport-Vassiliadis (RV) broth and incubated at 42 °C for 24 h. Then, the broths were subcultured on xylose-lysine-desoxycholate (XLD) agar (Oxoid) and incubated at 37 °C for 24 h. Next, the presumptive colonies were picked and subjected to standard biochemical methods (urea hydrolysis, H2S production on triple sugar iron agar, lysine decarboxylation, indole, methyl red test, Voges-Proskauer test and citrate utilization test). Typical *Salmonella* isolates were serotyped by slide agglutination test based on O and H antigens using polyvalent and monovalent antisera (DENKA SEIKEN Co., Japan) following the White-Kauffmann- Le Minor scheme [37].

### The in vitro sensitivity of *Salmonella* isolates to antimicrobial agents

Antibiograms of all identified *Salmonella* isolates were determined by the disc diffusion assay according to the guideline of Clinical and Laboratory Standards Institute [38] using Mueller-Hinton agar (Oxoid, Basingstoke, Hampshire, England, UK). Antimicrobial agents commonly used in either human or veterinary medicine was tested as follows: AMC (20/10 μg), AMP (10 μg), CEC (30 μg), CTX (30 μg), FEP (30 μg), DO (30 μg), CIP (5 μg), IPM (10 μg), S (10 μg), C (30 μg) and SXT (1.25/ 23.75 μg). All drugs were purchased from (Oxoid, England). *Escherichia coli* American Type Culture Collection (ATCC) 25922 were used as a reference strain. The isolates resistant to three or more separate classes of antimicrobials were defined as MDR [39]. Also, the MARI index was calculated for all *Salmonella* isolates according to the protocol designated by Krumperman [40] using the formula a/b (where “a” is the number of antimicrobials to which an isolate was resistant and “b” is the total number of antimicrobials to which the isolate was exposed).

### Molecular detection of *Salmonella* virulence-associated genes

The determination of *Salmonella* virulence factors was performed using the uniplex polymerase chain reaction assays targeting the *invA, csgD, hilC, bcfC, stn, avrA, mgtC, ompF, sopE1* and *pefA* gene sequences. DNA was extracted from 200 μl bacterial sample using a commercial kit (QIAamp DNA Mini kit, Qiagen, Germany) following the manufacturer’s instructions, and then stored at − 20 °C until further use. DNA concentration was measured by using a NanoDropTMND-1000 Spectrophotometer (Erlangen, Germany). Primer sequences and the expected size of the PCR product are detailed in Additional file 2: Table S1. All PCR reactions were performed using EmeraldAmp Max PCR Master Mix (Takara, Japan) in a final volume of 25 μl containing 12.5 μl of EmeraldAmp Max PCR Master Mix, 1 μl of each primer of 20 pmol concentrations, 4.5 μl of water, and 6 μl of DNA template. The Applied biosystem 2720 thermal cycler was programmed with specific profiles (Additional file 2: Table S1). Gel electrophoresis of the PCR products was applied to 1.5% agarose gel (Applichem, Germany). Next, the products were stained with ethidium bromide (Sigma-Aldrich, U.S.A.) and visualized under ultraviolet light photographed by a gel documentation system (Alpha Innotech, Biometra). Consideration of the positive result was depending on detection of a band similar to that in size of the positive control for a particular gene. Bacterial strains of *Salmonella* Enteritidis (ATCC 13076) were used as positive control for all PCR reactions.

### Statistical analysis

The obtained data were statistically analyzed using Pearson’s chi-square exact test using the SPSS Statistics 17.0 software program. The results were considered to be significant at *P* < 0.05.

## Additional files

Additional file 1: **Figure S1.** Amplification of (A) *invA* gene (651 bp) in *Salmonella* isolates. Lane L: DNA ladder (100 bp), lane Pos: Positive control, lane Neg: negative control, lanes 1–10: *invA* positive. (B) *csgD* gene (651 bp), lanes 1–5,7–10: *csgD* positive, lane 6: *csgD* negative. (C) *hilC* gene (241 bp) lanes 2,4–8: *hilC* positive, lanes 1,3,9,10: *hilC* negative. (D) *Stn* gene (617 bp) lanes 2,3,7,8: *stn* positive, lanes 1, 4–6,9,10: *stn* negative. **Figure S2.** Amplification of (A) *bcfC* gene (467 bp) in *Salmonella* isolates. Lane L: DNA ladder (100 bp), lane Pos: Positive control, lane Neg: negative control, lanes 2,3,7,8: *bcfC* positive, lanes 1,4-6,9,10: *bcfC* negative. (B) *mgtC* gene (677 bp) lanes 2,3,7: mgtC positive, lanes 1,4-6,8–10: *mgtC* negative. (C) *avrA* gene (422 bp) lanes 2,3,7: *avrA* positive, lanes 1,4-6,8–10: *avrA* negative. (D) *ompF* gene (519 bp) lanes 2,3: *ompF* positive, lanes 1,4–10: *ompF* negative. **Figure S3.** Amplification of (A) *sopE1* gene (422 bp) in *Salmonella* isolates. Lane L: DNA ladder (100 bp), lane Pos: Positive control, lane Neg: negative control, lanes 1–10: sopE1 negative (B) pefA gene (700 bp), lanes 1–10: pefA negative. (PDF 856 kb)

Additional file 2: **Table S1.** Primers sequences, target genes, amplicon sizes and cycling conditions for virulence factors [41–44]. **Table S2.** Distribution of virulence genes combinations and antibiotic resistance patterns in the different *Salmonella* serovars. (PDF 143 kb)

## Abbreviations

AMC: Amoxicillin-clavulanic acid
AMP: Ampicillin
ATCC: American Type Culture Collection
BPW: Difco-buffered peptone water
C: Chloramphenicol
CEC: Cefaclor
CIP: Ciprofloxacin
CTX: Cefotaxime
DO: Doxycycline
FEP: Cefepime
IPM: Imipenem
MARI: Multidrug antibiotic resistance index
MDR: Multidrug resistance
*pef*: Plasmid-encoded fimbriae
RV: Rappaport-Vassiliadis
S: Streptomycin
S.: *Salmonella*
SPIs: *Salmonella* pathogenicity islands
SXT: Trimethoprim sulfamethoxazole
XLD: Xylose-lysine-desoxycholate
