# Supplementary material for: Antimicrobial resistance profiles and virulence genotyping of Salmonella enterica serovars recovered from broiler chickens and chicken carcasses in Egypt
Source: BMC Vet Res. 2019 Apr 27;15:124. doi: 10.1186/s12917-019-1867-z (PMC6486964; doi:10.1186/s12917-019-1867-z)
Supplement: Supplementary file 1 — Figure S1. Amplification of (A) invA gene (651 bp) in Salmonella isolates. Lane L: DNA ladder (100 bp), lane Pos: Positive control, lane Neg: negative control, lanes 1–10: invA positive. (B) csgD gene (651 bp), lanes 1–5,7–10: csgD positive, lane 6: csgD negative. (C) hilC gene (241 bp) lanes 2,4–8: hilC positive, lanes 1,3,9,10: hilC negative. (D) Stn gene (617 bp) lanes 2,3,7,8: stn positive, lanes 1, 4–6,9,10: stn negative. Figure S2. Amplification of (A) bcfC gene (467 bp) in Salmonella isolates. Lane L: DNA ladder (100 bp), lane Pos: Positive control, lane Neg: negative control, lanes 2,3,7,8: bcfC positive, lanes 1,4-6,9,10: bcfC negative. (B) mgtC gene (677 bp) lanes 2,3,7: mgtC positive, lanes 1,4-6,8–10: mgtC negative. (C) avrA gene (422 bp) lanes 2,3,7: avrA positive, lanes 1,4-6,8–10: avrA negative. (D) ompF gene (519 bp) lanes 2,3: ompF positive, lanes 1,4–10: ompF negative. Figure S3. Amplification of (A) sopE1 gene (422 bp) in Salmonella isolates. Lane L: DNA ladder (100 bp), lane Pos: Positive control, lane Neg: negative control, lanes 1–10: sopE1 negative (B) pefA gene (700 bp), lanes 1–10: pefA negative. (PDF 856 kb) [file 12917_2019_1867_MOESM1_ESM.pdf]

Supplementary files

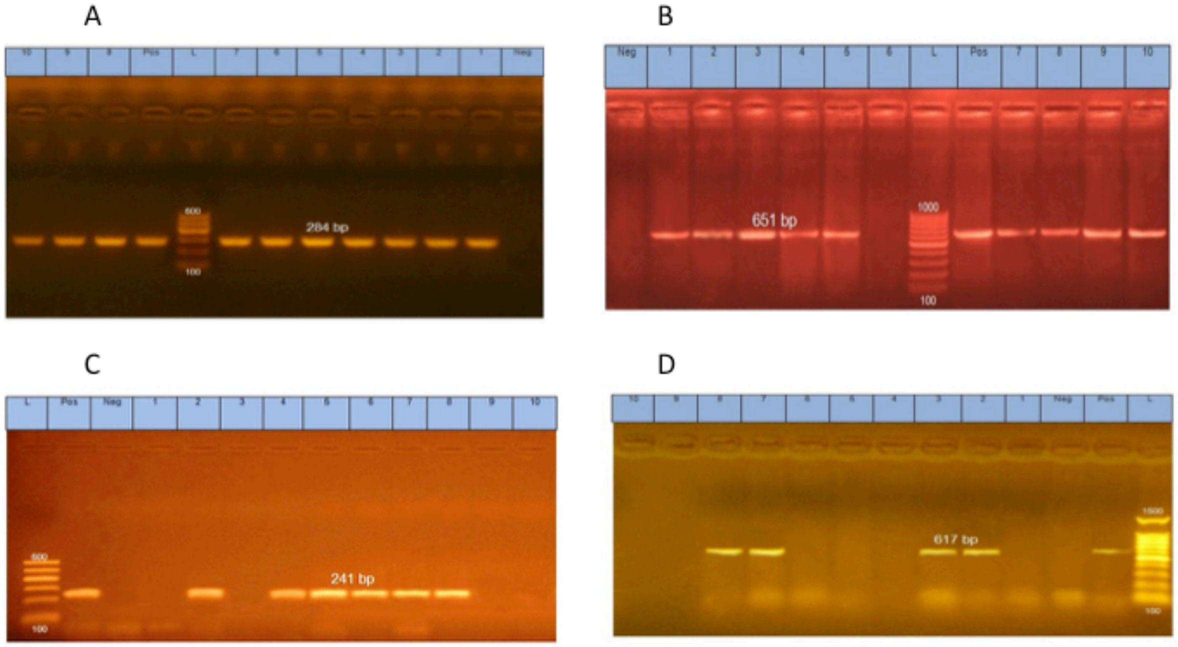

Figure S1. Amplification of **(A)** *invA* gene (651 bp) in *Salmonella* isolates. Lane L: DNA ladder (100 bp), lane Pos: Positive control, lane Neg: negative control, lanes 1-10: *invA* positive. **(B)** *csgD* gene (651 bp), lanes 1-5,7-10: *csgD* positive, lane 6: *csgD* negative. **(C)** *hilC* gene (241 bp) lanes 2,4-8: *hilC* positive, lanes 1,3,9,10: *hilC* negative. **(D)** *Stn* gene (617 bp) lanes 2,3,7,8: *stn* positive, lanes 1, 4-6,9,10: *stn* negative.

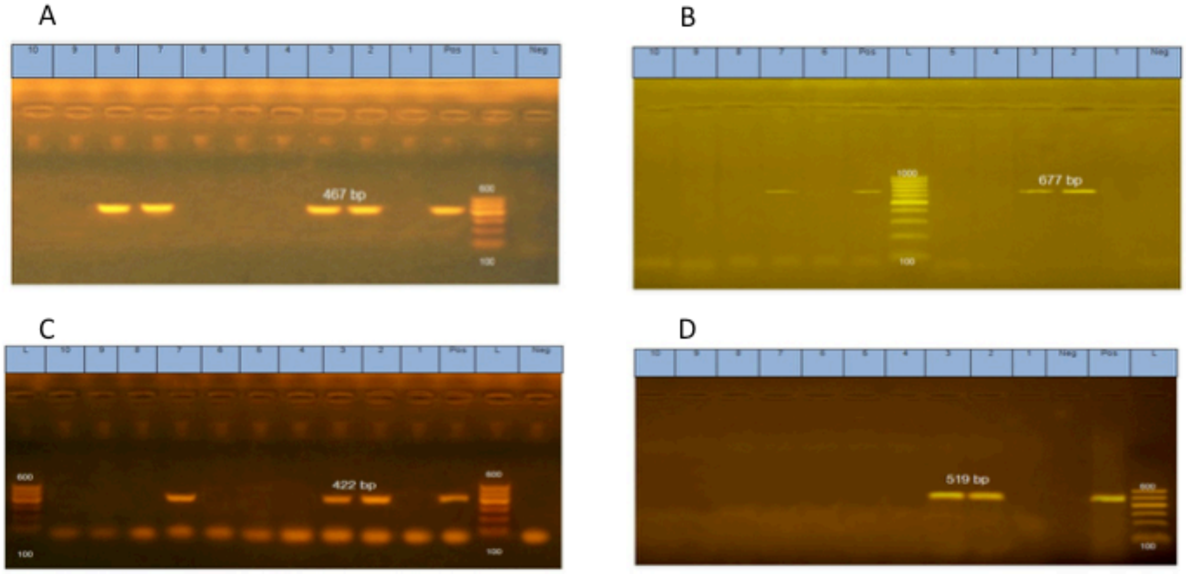

Figure S2. Amplification of **(A)** *bcfC* gene (467 bp) in *Salmonella* isolates. Lane L: DNA ladder (100 bp), lane Pos: Positive control, lane Neg: negative control, lanes 2,3,7,8: *bcfC* positive, lanes 1,4-6,9,10: *bcfC* negative. **(B)** *mgtC* gene (677 bp) lanes 2,3,7: mgtC positive, lanes 1,4-6,8-10: *mgtC* negative. **(C)** *avrA* gene (422 bp) lanes 2,3,7: *avrA* positive, lanes 1,4-6,8-10: *avrA* negative. **(D)** *ompF* gene (519 bp) lanes 2,3: *ompF* positive, lanes 1,4-10: *ompF* negative.

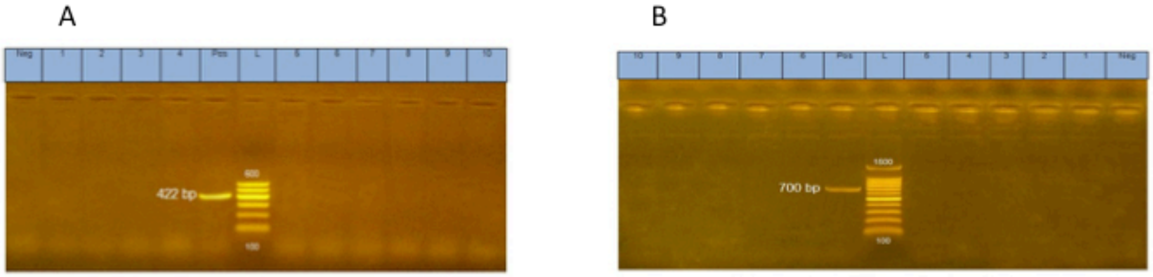

Figure S3. Amplification of **(A)** *sopE1* gene (422 bp) in *Salmonella* isolates. Lane L: DNA ladder (100 bp), lane Pos: Positive control, lane Neg: negative control, lanes 1-10: sopE1 negative **(B)** pefA gene (700 bp), lanes 1-10: pefA negative.
