# Supplementary material for: Antimicrobial resistance profiles and virulence genotyping of Salmonella enterica serovars recovered from broiler chickens and chicken carcasses in Egypt
Source: BMC Vet Res. 2019 Apr 27;15:124. doi: 10.1186/s12917-019-1867-z (PMC6486964; doi:10.1186/s12917-019-1867-z)
Supplement: Supplementary file 2 — Table S1. Primers sequences, target genes, amplicon sizes and cycling conditions for virulence factors [41–44]. Table S2. Distribution of virulence genes combinations and antibiotic resistance patterns in the different Salmonella serovars. (PDF 143 kb) [file 12917_2019_1867_MOESM2_ESM.pdf]

Supplementary files

Table S1. Primers sequences, target genes, amplicon sizes and cycling conditions for virulence factors

Gene name	Location/Function	Primers sequences	Amplified segment (bp)	Primary denaturation	Amplification (35 cycles)			Final extension	Reference
					Secondary denaturation	Annealing	Extension		
invA	*SPI-1/Invasion of macrophages	GTGAAATTATCGCCACGTTCG GGCAA	284	94˚C 5 min.	94˚C 30 sec.	55˚C 30 sec.	72˚C 30 sec.	72˚C 7 min.	Oliveira et al. [6]
csgD	Chromosome/master regulator of the biofilm matrix compounds	TTACCGCCTGAGATTATCGT<br>ATGTTTAATGAAGTCCATAG	651	94˚C 5 min.	94˚C 30 sec.	50˚C 45 sec.	72˚C 45 sec.	72˚C 10 min.	Bhowmick et al. [41]
MgtC	SPI-3/ Mg2+ uptake	TGA CTA TCA ATG CTC CAG TGA AT<br>ATT TAC TGG CCG CTA TGC TGT TG	677	94˚C 5 min.	94˚C 45 sec.	58˚C 45 sec.	72˚C 45 sec.	72˚C 10 min.	Huehn et al. [42]
bcfC	Chromosome/Bovine colonisation factor, fimbrial usher	ACC AGA GAC ATT GCC TTC C<br>TTC TGC TCG CCG CTA TTC G	467	94˚C 5 min.	94˚C 45 sec.	53˚C 45 sec.	72˚C 45 sec.	72˚C 10 min.	
sopE1	Cryptic bacteriophage/Promotes membrane ruffling and disrupts tight junctions	ACT CCT TGCACA ACC AAA<br>TGC GGA TGT CTTCTG CAT TTC GCC ACC	422	94˚C 5 min.	94˚C 45 sec.	58˚C 45 sec.	72˚C 45 sec.	72˚C 10 min.	
avrA	SPI-1/Controls *Salmonella*induced	CCT GTA TTG TTG AGC GTC TGG	422	94˚C 5 min.	94˚C 45 sec.	58˚C 45 sec.	72˚C 45 sec.	72˚C 10 min.	

	Inflammation	AGA AGA GCT TCG TTG AAT GTC C								
pefA	Plasmid/Plasmid encoded fimbriae	TGT TTC CGG GCT TGT GCT	700	94˚C 5 min.	94˚C 45 sec.	55˚C 45 sec.	72˚C 45 sec.	72˚C 10 min.	Murugkar et al. [43]
		CAG GGC ATT TGC TGA TTC TTC C								
Stn	Chromosome/ Enterotoxin	TTG TGT CGC TAT CAC TGG CAA CC	617	94˚C 5 min.	94˚C 45 sec.	59˚C 45 sec.	72˚C 45 sec.	72˚C 10 min.		
		ATT CGT AAC CCG CTC TCG TCC								
hilC	SPI-1/ modulate invasion gene expression	GGACTTGTTGCCAGGGATG	241	94˚C 5 min.	94˚C 30 sec.	62˚C 30 sec.	72˚C 30 sec.	72˚C 7 min.	Yang et al. [44]
		TGACCATTTGCGGGTGAG								
ompF	Chromosome/ Outer membrane porins	CCTGGCAGCGGTGATCC	519	94˚C 5 min.	94˚C 45 sec.	50˚C 45 sec.	72˚C 45 sec.	72˚C 10 min.	Tatavarthy and Cannons [23]
		TGGTGTAACCTACGCCATC								

*SPI: *Salmonella* Pathogenicity Island

Table S2. Distribution of virulence genes combinations and antibiotic resistance patterns in the different *Salmonella* serovars

**Serovars (n)**	**Sample**	**Antibiotic resistance (pattern profile)**	**Genetic profile**	**Virulence genes**
S. Enteritidis (2)	Cloacal swabs	SXT,AMP,AMC,CEC,FEP,CTX	P2	*invA, csgD, bcfC, stn, avrA, mgtC, ompF*
S. Enteritidis (2)	Cloacal swabs	SXT, AMP,S,DO,CIP	P2	*invA, csgD, bcfC, stn, avrA, mgtC, ompF*
S. Enteritidis (2)	Cloacal swabs	SXT,AMC	P4	*invA, csgD, hilC, bcfC, stn*
S. Enteritidis (2)	Cloacal swabs	SXT, AMP,AMC,S, DO,CEC	P4	*invA, csgD, hilC, bcfC, stn*
S. Enteritidis (2)	Cloacal swabs	SXT, AMP,AMC,S,DO	P5	*invA, csgD, hilC*
S. Enteritidis (4)	Cloacal swabs	SXT, AMP,S,DO,CIP	P5	*invA, csgD, hilC*
S. Enteritidis (6)	Cloacal swabs	SXT, AMP,AMC,S,DO	P3	*invA, csgD, hilC, bcfC, stn, avrA, mgtC*
S. Enteritidis (6)	Water	SXT, AMP,AMC,S, DO,CEC,C	P1	*invA, csgD, hilC, bcfC, stn, avrA, mgtC, ompF*
S. Enteritidis (4)	Feed	SXT,AMC	P6	*invA, hilC*
S. Enteritidis (2)	Carcass	SXT,AMP,AMC,S,CEC,C,FEP	P1	*invA, csgD, hilC, bcfC, stn, avrA, mgtC,ompF*
S. Enteritidis (2)	Carcass	SXT, AMP,AMC,S,DO	P1	*invA, csgD, hilC, bcfC, stn, avrA, mgtC,ompF*
S. Enteritidis (2)	Carcass	SXT, AMP,AMC,S, DO,CEC,C	P3	*invA, csgD, hilC, bcfC, stn, avrA, mgtC*
S. Enteritidis (4)	Carcass	SXT,AMP,AMC,CEC,FEP,CTX	P3	*invA, csgD, hilC,bcfC,stn, avrA, mgtC*

S. Enteritidis (8)	Carcass	SXT,AMC	P7	*invA, csgD*
*S.*Typhimurium (10)	Cloacal swabs	SXT,S, DO,CEC	P6	*invA, hilC*
*S.*Typhimurium (9)	Cloacal swabs	SXT,AMC	P7	*invA, csgD*
*S.*Typhimurium (4)	Feed	SXT,AMP,AMC,C	P6	*invA, hilC*
*S.*Typhimurium (2)	Letter	SXT,AMP,AMC,S,DO,CTX,CIP	P2	*invA, csgD, bcfC, stn, avrA, mgtC, ompF*
*S.*Typhimurium (3)	Carcass	SXT,S, DO,CEC	P2	*invA, csgD, bcfC, stn, avrA, mgtC, ompF*
S. Typhimurium (2)	Carcass	SXT,AMP,AMC,S,CEC,C,FEP	P3	*invA, csgD, hilC, bcfC, stn, avrA, mgtC*
*S.*Typhimurium(2)	Carcass	SXT, AMP,AMC,S, DO,CEC	P4	*invA, csgD, hilC, bcfC, stn*
*S.*Typhimurium (1)	Carcass	SXT,AMP,S	P4	*invA, csgD, hilC, bcfC, stn*
*S.*Typhimurium (2)	Carcass	SXT,AMP,AMC,CEC,FEP,CTX	P5	*invA, csgD, hilC*
*S.*Typhimurium	Carcass	SXT,S, DO,CEC	P6	*invA, hilC*
*S.*Kentucky	Cloacal swabs	SXT, AMP,AMC,S,DO	P7	*invA, csgD*
*S.*Kentucky (2)	Carcass	SXT, AMP,AMC,S, DO,CEC,C	P5	*invA, csgD, hilC*
*S.*Kentucky (2)	Carcass	SXT,AMP,S	P7	*invA, csgD*
*S.*Kentucky (2)	Carcass	SXT,AMP,AMC,CEC,FEP,CTX	P7	*invA, csgD*
*S.*Kentucky	Carcass	SXT,AMP,AMC,S,DO,CTX,CIP	P5	*invA, csgD, hilC*

*S.*Kentucky	Carcass	SXT,AMP,AMC,S,DO,CTX,CIP	P5	*invA, csgD, hilC*
*S.*Kentucky (3)	Fan swabs	SXT, AMP,AMC,S,DO	P7	invA, csgD
*S.*Molade (6)	Cloacal swabs	SXT,AMP,S	P5	invA, csgD, hilC
*S.*Molade (2)	Carcass	SXT, AMP,AMC,S, DO,CEC	P7	invA, csgD
*S.*Molade (4)	Carcass	SXT,AMC	P7	invA, csgD
S. Bargny (2)	Carcass	SXT,AMP,S	P5	invA, csgD, hilC
S. Bargny (2)	Cloacal swab	SXT,AMP,S	P5	invA,csgD, hilC
S. Bargny (2)	Water	SXT,AMC	P7	invA, csgD
S. Inganda (4)	Carcass	SXT,AMP,S	P7	invA, csgD
S. Infantis	Carcass	SXT,AMP,S	P7	invA, csgD
S. Infantis	Workers hand swabs	SXT,AMP,S	P7	invA, csgD

AMC=Amoxicillin-clavulanic acid, AMP=ampicillin, CEC=Cefaclor, CTX=Cefotaxime, FEP=Cefepime, DO=Doxycycline, CIP=Ciprofloxacin, IPM=Imipenem, S=Streptomycin, C=Chloramphenicol, and SXT= trimethoprim sulfamethoxazole.
